# Metabolomic Profiling of Erector Spinae Plane Block for Breast Cancer Surgery

**DOI:** 10.3390/medicina61071294

**Published:** 2025-07-18

**Authors:** Ekin Guran, Ozan Kaplan, Serpil Savlı, Cigdem Sonmez, Lutfi Dogan, Suheyla Unver

**Affiliations:** 1 Department of Anesthesiology and Reanimation, Ankara Oncology Training and Research Hospital, The University of Health Sciences, Ankara, TR 06100, USA; ssavli@yahoo.com (S.S.); sunver2@yahoo.com (S.U.); 2 Department of Analytical Chemistry, Faculty of Pharmacy, Hacettepe University, Ankara, TR 06800, USA; ozankaplan@hacettepe.edu.tr; 3 Department of Medical Biochemistry, Ankara Oncology Training and Research Hospital, The University of Health Sciences, Ankara, TR 06100, USA; dr.csonmez@gmail.com; 4 Department of Surgical Oncology, Ankara Oncology Training and Research Hospital, The University of Health Sciences, Ankara, TR 06100, USA; lutfidogan1@yahoo.com

**Keywords:** metabolomics, vascular endothelial growth factor, erector spinae plane block, breast cancer

## Abstract

*Background and Objectives:* Regional and systemic analgesic techniques, such as erector spinae plane (ESP) block and opioid administration, implemented during cancer surgery, have been shown to influence immune responses and potentially affect cancer outcomes. Surgical stress and analgesic techniques used in cancer surgery—such as regional nerve blocks or systemic opioids—not only affect pain control but also influence immune and inflammatory pathways that may impact cancer progression. To understand the biological consequences of these interventions, metabolomic profiling has emerged as a powerful approach for capturing systemic metabolic and immunological changes, which are particularly relevant in the oncologic perioperative setting. In this study, we examined the impact of the ESP on the metabolomic profile, as well as levels of VEGF, cortisol, and CRP, in addition to its analgesic effects in breast cancer surgery. *Materials and Methods:* Ninety patients were placed into three different analgesia groups (morphine, ESP, and control groups). Demographic data, ASA classification, comorbidities, surgery types, and pain scores were documented. Blood samples were taken at preoperative hour 0, postoperative hour 1, and postoperative hour 24 (T0, T1, and T24). VEGF, cortisol, and CRP levels were measured, and metabolomic analysis was performed. *Results:* Study groups were comparable regarding demographic findings, comorbidities, and surgery types (*p* > 0.05). NRS scores of group ESP were lowest in the first 12 h period (*p* < 0.01) and ESP block reduced opioid consumption (*p* < 0.01). VEGF and cortisol levels of group morphine were similar to ESP at T24 (*p* > 0.05). Group ESP had lower VEGF and cortisol levels than the control at T24 (*p* = 0.025, *p* = 0.041, respectively.). The CRP level of group morphine was higher than both ESP and control at T24 (*p* = 0.022). Metabolites involved in primary bile acid, steroid hormone biosynthesis, amino acid, and glutathione metabolism were changed in group ESP. *Conclusions:* Metabolites in bile acid biosynthesis and steroid hormone pathways, which play a key role in immune responses, were notably lower in the ESP group. Accordingly, VEGF and cortisol peaks were more moderate in group ESP. In conclusion, we think that ESP block, which provides adequate analgesia, is an acceptable approach in terms of modulating immune responses in breast cancer surgery.

## 1. Introduction

Breast cancer is the most commonly diagnosed malignancy among women and remains a leading cause of cancer-related mortality worldwide [1]. Curative surgery is the mainstay of treatment in early-stage disease [2]. However, despite curative intent, surgical trauma and perioperative stress responses may contribute to tumor recurrence and metastasis by promoting immunosuppression, inflammation, and angiogenesis [3]. The perioperative period has been shown to affect the tumor immune microenvironment. Surgical trauma increases growth factors and catecholamines due to sympathetic signaling and immunosuppression thus allowing cancer cells to evade detection by the immune system [4,5,6]. There is increasing evidence that the combined results of these changes may enhance the viability of circulating tumor cells and lead to perioperative tumor growth [3,7,8]. Given the susceptibility to tumor progression during the perioperative period, it is imperative to prioritize strategies that sustain immune responses [9,10]. The perioperative period has thus been termed a “window of opportunity”, during which interventions aimed at modulating the immune response may significantly impact long-term oncological outcomes. There are three key pathways potentially linked to cancer recurrence during the perioperative period: inflammation, immunosuppression, and angiogenesis [11]. The angiogenesis pathway can be assessed by vascular endothelial growth factor (VEGF), immunosuppression by cortisol, and inflammation by interleukin-6 or C-reactive protein (CRP) levels [12,13,14]. Higher VEGF levels following breast cancer surgery are associated with the recurrence of residual disease [15]. Regional anesthesia has been shown to lower cortisol levels and potentially reduce postoperative immunosuppression, though its significance on oncological outcomes remains uncertain [16]. Elevated postoperative CRP levels are associated with poor oncological outcomes, but the impact of analgesic applications on these results is unknown [17].

Metabolites are low-molecular-weight chemical molecules involved in cellular reactions. Metabolomics is the field dedicated to studying how these metabolites vary under specified conditions [18]. Metabolomic profiling in cancer surgery provides valuable insights into systemic cancer metabolism in the perioperative period [19]. However, metabolomic profiling of anesthetic techniques is a relatively new area. There are currently no studies that examine the impact of regional blocks on cancer metabolomics. Untargeted metabolomics enables an unbiased investigation of physiological shifts, potentially revealing novel biomarkers related to immune suppression or tumor-promoting processes. Despite its growing relevance, there is currently no study evaluating the metabolomic impact of regional anesthesia techniques such as ESP block in the oncological perioperative setting.

This study aims to investigate whether the ESP block modulates the postoperative immune microenvironment by evaluating metabolomic changes and immune-related biomarkers (VEGF, cortisol, CRP). We also aimed to assess its analgesic efficacy and opioid-sparing potential. We hypothesized that ESP block, beyond providing effective pain control, may exert immunomodulatory effects that are measurable through metabolomic and biochemical changes in the perioperative period.

## 2. Materials and Methods

### 2.1. Study Design

This prospective observational study was conducted at a high-volume oncology center. Ninty female patients with stage 1–2 primer unilateral breast cancer were included after Ankara Oncology Health Application and Research Center Ethics Committee approval (2020-12/898, NCT04689945). The surgeries were performed by the same experienced surgical oncologists to minimize variability in surgery-related inflammatory responses. Patients with a history of cancer or cancer treatment (surgery, chemotherapy, immunotherapy, etc.), chronic steroid treatment, smoking, coronary artery disease, chronic obstructive pulmonary disease, inflammatory breast cancer, and autoimmune disease were excluded. Weight, height, body mass index (BMI), American Society of Anesthesiologists (ASA) physical status, comorbidities, and surgery type were recorded.

Patients were placed into three groups and treatment regimen details are available in the Supplementary Materials. (group ESP, n = 30; morphine, n = 31; control, n = 29). Group control was formed to examine the analgesic efficacy of ESP block. Group control had the same multimodal analgesia regimen as Group ESP except for block application. USG-guided ESP blocks were performed at the T4 level before surgery (bupivacaine 0.25%, 20 mL). Propofol 2–3 mg/kg, fentanyl 1–2 mcg/kg, and rocuronium 0.6 mg/kg were administered for anesthesia induction and 2% sevoflurane, 40% O2, and 60% N20 were administered after intubation to all patients. BIS scores were kept between 40 and 60 during surgery. All patients received intravenous (iv) 1 gr paracetamol at the end of the surgery. Additionally, group morphine received 0.1 mg/kg iv. morphine, while the ESP and control groups received 50 mg dexketoprofen. Patient-controlled analgesia (PCA) devices were applied to all patients. Group morphine received morphine-PCA (1 mg/mL), while group ESP and control received tramadol-PCA (10 mg/mL). These patients are selected to separate the effect of 24 h morphine consumption in the metabolomic profile. NRS scores and opioid consumption were recorded at the postoperative 1, 2, 12, and 24th h. Blood samples were collected three times (preoperative = T0, postoperative hour 1 = T1, postoperative hour 24 = T24). CRP and cortisol levels were documented at all times. VEGF was recorded at T0 and T24 since VEGF peaks at least 12 h postoperatively [20] (ELISA, SUNREDBIO, Shanghai, China).

### 2.2. Metabolomic Analysis

Metabolomic analysis of group ESP and morphine was performed at T24 via an Agilent 6530 Quadrupole Time-of-Flight Mass Spectrometer (Q-TOF/MS), Agilent Technologies Inc., Santa Clara, CA, USA. First, samples that had been stored at −80 °C were allowed to reach room temperature. Next, 200 µL of plasma from the samples was taken into a microcentrifuge tube and metabolite extraction was performed by adding 600 µL of methanol. The vortexed mixture was centrifuged at 9000 rpm, +4 °C for 30 min and the supernatant metabolite phase was collected. The metabolite phase was evaporated by using a vacuum centrifuge and then redissolved in 200 µL of a 50:50 (*v*/*v*) mixture of Milli-Q water and acetonitrile. A quality control (or QC) sample, consisting of pooled aliquots from all samples, was prepared to assess method reproducibility. All solvents and chemicals used in sample preparation were obtained from Merck (Darmstadt, Germany) and were of at least MS grade or higher. The mobile phase, used with a C18 (2.1 × 100 mm × 2.5 µm, XBridge, Waters, Milford, MA, USA) chromatography column, consisted of water with 0.1% formic acid and acetonitrile with 0.1% formic acid. The device was operated in positive ion mode with a scan range of 100–1700 *m*/*z*. Samples were injected in random order, with a quality control sample run every six injections.

### 2.3. Statistical Analysis

Datasets were analyzed with the Statistical Package for Social Sciences for Windows 22.0 (SPSS Inc, Chicago, IL, USA). Normal distribution was assessed with the Q-Q plot test. Categorical variables were expressed as frequency and percentage, while continuous variables were reported as mean ± standard deviation (SD). Fisher’s Exact Test and Pearson chi-square tests were applied for categorical data, and Student’s *t*-test or one-way ANOVA was used for group comparisons, with statistical significance set at *p* < 0.05. For metabolomic data preprocessing, raw LC-MS data (.d format) were converted to .mzXML using ProteoWizard (version 3) and processed in MZmine (version 2.53)) for peak detection and alignment. A total of 2804 features were extracted. For normalization, each peak intensity was divided by the average intensity of all peaks within the same sample (i.e., per-sample mean normalization). This approach aimed to correct for inter-sample variation in total signal intensity. Student’s *t*-test was used for group comparison, with significance defined as *p* < 0.05 and fold change >2.0. Principal component analysis, volcano plot, heat map, and pathway analysis were conducted with MetaboAnalyst 5.0 [21]. Principal component analysis (PCA) was performed using the full matrix of raw metabolic features (peaks) detected in plasma samples from ESP and morphine groups at postoperative hour 24 (T24). Prior to PCA, all peak intensities were auto-scaled (mean-centered and divided by the standard deviation) to account for differences in magnitude among variables. Metabolomic pathway analysis was performed using the list of identified metabolites. These metabolites were subjected to pathway enrichment and topology analysis. Over-representation analysis (ORA) was conducted using the hypergeometric test, and pathway topology was evaluated based on relative betweenness centrality. The most significantly enriched pathways were ranked according to their FDR-adjusted *p*-values.

## 3. Results

### 3.1. Demographic Data of Participants

Ninety patients were included. Two patients were excluded from the study because of ESP block failure and the misuse of the PCA, and one was also excluded since she withdrew her consent, thus data from 87 patients were analyzed. Three groups were comparable in terms of age, body weight, height, BMI, ASA status, and comorbidities (*p* > 0.05). Surgery types of groups were similar (*p* > 0.05) and the most common type of surgery in all three groups was segmental mastectomy (64.3%) (Table 1). There were no statistically significant differences among the groups for any baseline demographic or surgical variables, confirming group comparability.

### 3.2. Analgesic Outcomes of Groups

Analgesic outcome refers to postoperative pain scores (Numeric Rating Scale, NRS: 0–10) at fixed time points (1, 2, 12, and 24 h), along with total opioid consumption over 24 h. Morphine-PCA was used in 29 patients and tramadol-PCA was used in 58 patients. T0 and T24 NRS scores were similar in three groups (*p* > 0.05). The NRS scores in group ESP at the 1st, 2nd, and 12th hours postoperatively were significantly lower (*p* < 0.01). Group morphine consumed 11.1 ± 4.1 mg of morphine in 24 h, while group ESP consumed 57 ± 38 mg, and the control group consumed 166 ± 69 mg of tramadol. Tramadol consumption was significantly lower in the ESP block group (*p* < 0.01) (Table 2).

### 3.3. VEGF, Cortisol, and CRP Levels of Groups

The VEGF levels were comparable in three groups at T0 (*p* > 0.05). VEGF levels of group ESP were similar to group morphine but significantly lower than the group control at T24 (*p* = 0.025) Cortisol levels were similar between the three groups at T0 and T1 (*p* > 0.05). Also, they were similar between ESP and morphine in T24 (*p* > 0.05) while they were lower in group ESP compared to group control (*p* = 0.041). T0 CRP levels were similar in all three groups (*p* > 0.05). CRP levels of group morphine were significantly higher than group ESP at T1 (*p* = 0.028). At T24, the CRP level of group morphine was significantly higher than the other two groups (*p* = 0.022) (Table 3).

### 3.4. Metabolomic Profiles of ESP and Morphine Groups

**Figure 1:**
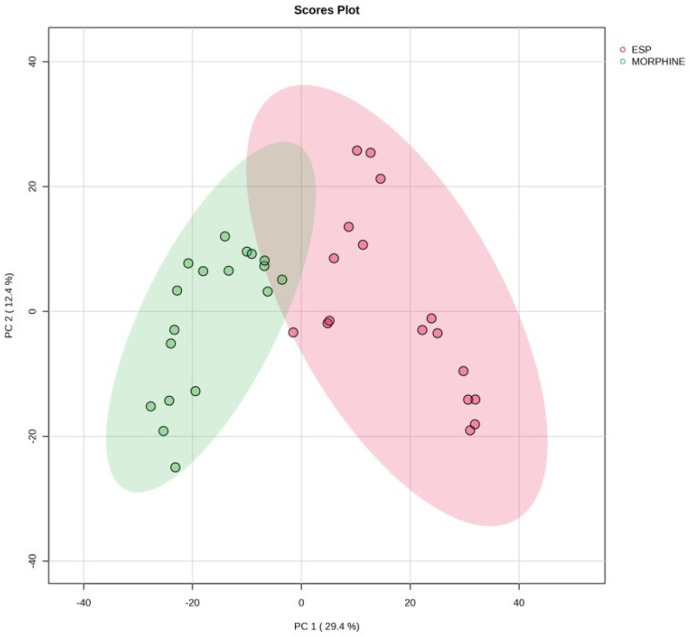
Principal component analysis of groups morphine and ESP at postoperative 24th hour.

While the biochemical analysis included all participants, the metabolomic profiles at T24 were specifically compared between the ESP and morphine groups to assess the effects of morphine-based versus ESP block-based analgesia. In total, 2804 common peaks were identified between groups ESP and morphine, with 1001 showing statistical significance (*p* < 0.05). Principal component analysis demonstrated a clear metabolic separation between the ESP and morphine groups, indicating a distinct metabolomic response to the analgesic modality used (Figure 1).

**Figure 2:**
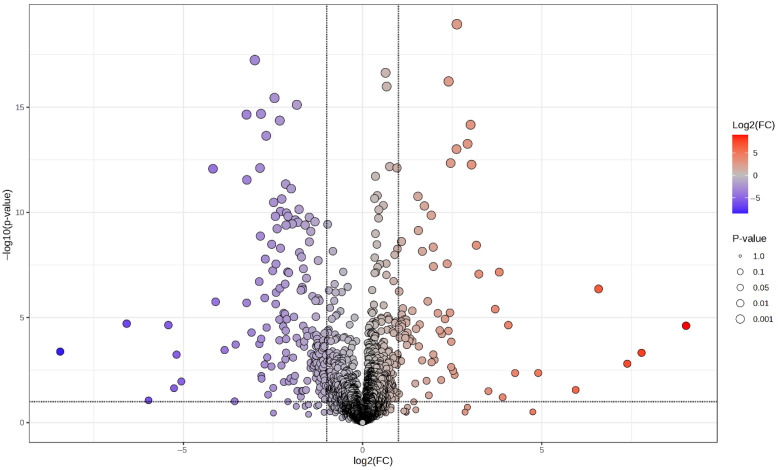
The volcano plot graph of the peaks that changed significantly between groups ESP and morphine.

We created a volcano plot to show significant peaks differences between the two groups (Figure 2). Elevated metabolites in group ESP are shown as red circles, and decreased levels are in blue circles. A total of 152 peaks matched metabolites in the in-house library, with 84 peaks significantly differing between the two groups (Table 4). This table includes log2 (fold change), *p* values, and biological classes of metabolites, highlighting the differences between ESP and morphine.

**Figure 3:**
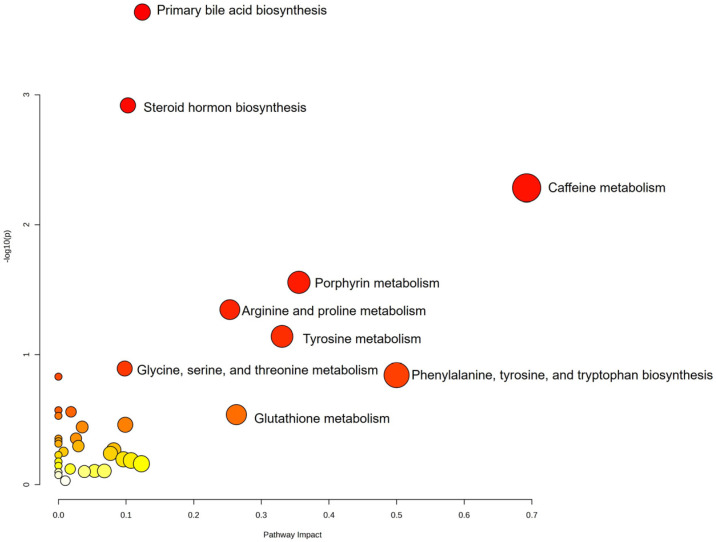
The most altered pathways between groups ESP and morphine.

Pathway analysis provides an overview of pathways linked to matching metabolites, using color-coded circles based on *p*-values: red for the most comparisons and lighter colors for fewest. After excluding pathways related to 84 metabolites with significant changes, the most altered pathways are primary bile acid biosynthesis, steroid hormone biosynthesis, porphyrin metabolism, amino acid metabolism, and glutathione metabolism (Figure 3).

## 4. Discussion

Surgical excision remains a cornerstone in the curative treatment of early-stage breast cancer [1,2]. However, the perioperative period has increasingly been recognized as a critical immunological window that may influence cancer recurrence due to inflammatory responses, immunosuppression, and angiogenesis [3,4,5,6]. Minimally invasive surgeries such as breast-conserving procedures are associated with improved cosmetic outcomes and equivalent oncological safety [22], yet surgical trauma even in limited procedures triggers a cascade of neuroendocrine stress responses that can compromise host anti-tumor immunity [4,5,23,24,25].

Anesthetic techniques can modify this perioperative immune landscape. Compared to general anesthesia alone, regional techniques like paravertebral block have been associated with reduced risk of cancer recurrence or metastasis [26,27]. This benefit may stem from transient chemical sympathectomy, reduced opioid use, and direct antiproliferative effects of local anesthetics [28,29]. The ESP block, a relatively newer regional technique, has shown promise in reducing postoperative opioid consumption while providing effective analgesia in breast cancer surgery [30,31].

In this study, ESP block significantly reduced pain scores in the early postoperative period and lowered tramadol consumption compared to control. However, pain scores at 24 h were similar across groups, likely due to the diminishing analgesic effect of a single ESP block and overlapping systemic analgesia [31]. Beyond pain control, the ESP block may influence immune pathways by modulating sympathetic output [32], although its exact immunological impact remains under investigation. A prior study suggested that ESP may be less immunomodulatory than paravertebral block [33], underscoring the need for further mechanistic data.

Our findings demonstrate that ESP block attenuated increases in serum VEGF, cortisol, and CRP levels, which are markers associated with poor oncological outcomes [13,34,35,36,37,38]. While VEGF levels at T0 were comparable, group ESP showed significantly lower levels than the control group at T24 (*p* = 0.025). VEGF is known to stimulate angiogenesis and suppress anti-tumor immunity [34,35,36], and its elevation following tumor resection may promote recurrence [13]. The difference between the ESP and control groups may be attributed to chemical sympathectomy via β-adrenergic inhibition by bupivacaine [39] or the immunomodulatory effects of lower tramadol consumption [40,41].

Similarly, cortisol, a hallmark of surgical stress, was significantly lower in the ESP group at T24 compared to control. Cortisol elevation promotes immunosuppression and tumor progression [42]. Our findings align with prior reports suggesting regional anesthesia dampens cortisol release more effectively than general anesthesia alone [43], and ESP may be superior to paravertebral block in this regard [33]. Notably, metabolomic profiling also revealed lower levels of 21-deoxycortisol in the ESP group, reinforcing the biochemical correlation with stress modulation.

CRP, beyond being a passive marker of inflammation, is an active participant in immune signaling through pathways such as complement activation and phagocytosis [37]. Elevated CRP has been associated with poor prognosis following cancer surgery [38]. In our study, CRP levels were significantly higher in the morphine group compared to ESP at both T1 and T24, suggesting that opioid-based analgesia may exacerbate postoperative inflammation.

The differential immunological profiles between the ESP and morphine groups were further elucidated through untargeted metabolomics. We identified 84 significantly altered metabolites involved in pathways such as amino acid metabolism, bile acid biosynthesis, steroid hormone biosynthesis, prostaglandin signaling, porphyrin metabolism, and glutathione metabolism.

Morphine has been shown to suppress natural killer cell activity [44], promote angiogenesis [45], and support tumor growth [46], while polymorphisms in the µ-opioid receptor may impact breast cancer survival [47]. In contrast, tramadol appears to enhance immune responses, including natural killer cell function and lymphocyte proliferation, potentially via serotonergic mechanisms [40,48,49]. It has been associated with reduced interleukin-6 and tumor necrosis factor-α levels and anti-tumor activity in breast cancer xenograft models [44,50]. These immunomodulatory properties may partially explain the favorable biomarker and metabolite profile observed in the ESP group.

Among the most altered pathways, bile acid metabolism drew particular attention. Several bile acid derivatives including glycocholate, taurocholate, and taurochenodeoxycholate were significantly elevated in the ESP group. Bile acids influence the tumor immune microenvironment, with certain metabolites inhibiting breast cancer cell proliferation and correlating with improved survival [51,52,53]. Nonetheless, bile acids can also exert pro-tumorigenic effects depending on receptor context and concentration, necessitating careful interpretation.

Steroid hormone biosynthesis also showed distinct patterns. The ESP group exhibited elevated 20α-dihydroprogesterone, which is a progesterone metabolite known to downregulate endothelial inflammatory markers [54]. This suggests a potential anti-inflammatory microenvironment fostered by ESP block, which may be especially relevant in hormone-sensitive tumors [55,56,57,58].

The ESP group also demonstrated reduced levels of porphyrins and heme-related compounds (Coproporphyrin I/III, heme), which are linked to oxidative stress and inflammation [59,60]. However, heme plays a dual role in cancer: while its reduction may suppress inflammation, it is also implicated in ferroptosis a form of programmed cell death leveraged in triple-negative breast cancer therapy [61].

Amino acid metabolism also differed significantly. Arginine and tyrosine, which are both elevated in the ESP group, are crucial to immune cell proliferation and stress response [62,63]. Although elevated tyrosine may suggest heightened catecholamine turnover, the concomitant reduction in cortisol implies a well-regulated stress response rather than sympathetic overactivation.

Finally, glutathione metabolism was enhanced in the ESP group. While this suggests increased antioxidant defense, it may paradoxically attenuate ferroptotic tumor cell death, particularly in glutathione-driven subtypes of triple-negative breast cancer [64,65]. Whether this confers benefit or risk remains unclear.

To our knowledge, this is the first study to examine the immunometabolic consequences of ESP block in breast cancer surgery using combined biochemical and untargeted metabolomic analyses. The inclusion of treatment-naïve stage I–II patients allows for unbiased interpretation of immune dynamics without interference from adjuvant therapy.

This study has limitations. The observational design and modest sample size restrict generalizability. The short follow-up precludes conclusions on long-term oncologic outcomes. Moreover, attributing immune changes solely to ESP block is challenging due to the multimodal nature of analgesic regimens.

Nonetheless, our findings suggest that ESP block may favorably modulate perioperative immune responses, reduce pro-tumorigenic signaling, and shift the metabolic landscape toward less inflammatory profiles. These data support the integration of regional techniques like ESP into multimodal analgesia regimens in breast cancer surgery—not just for pain control, but also for their potential systemic immunologic benefits.

## 5. Conclusions

In this prospective observational study, we investigated the immunometabolic ESP block in breast cancer surgery using clinical, biochemical, and metabolomic analyses. Our findings indicate that ESP block not only provides effective postoperative analgesia but may also exert favorable systemic effects on the immune and metabolic landscape in the critical perioperative period. Compared to both systemic morphine and control groups, the ESP group demonstrated significantly lower levels of VEGF, cortisol, and CRP at 24 h postoperatively. These biomarkers are well-established indicators of angiogenesis, immunosuppression, and systemic inflammation, all of which contribute to an immunological environment conducive to tumor recurrence. The attenuation of these markers in the ESP group suggests that regional anesthesia may offer protection against the perioperative immune suppression commonly associated with surgical stress. Metabolomic profiling further revealed that ESP block induced distinct changes in several key pathways, including bile acid biosynthesis, steroid hormone metabolism, porphyrin metabolism, glutathione metabolism, and amino acid biosynthesis. The elevation of anti-inflammatory bile acid derivatives and progesterone metabolites, alongside reduced levels of heme and porphyrins, supports the hypothesis that ESP block may create a less inflammatory, more regulated immune microenvironment. These metabolic shifts reinforce the biochemical data and highlight the potential of ESP block to modulate systemic immunity beyond its analgesic role. Importantly, this study included only treatment-naïve stage I–II breast cancer patients, which allowed for a clearer examination of perioperative immune modulation in the absence of prior chemotherapy, radiotherapy, or hormonal treatment. This strengthens the internal validity of our findings and offers a more accurate representation of how regional anesthesia techniques interact with innate immune physiology in the surgical context. Despite these promising findings, our study has limitations. The observational design and relatively short follow-up period preclude conclusions regarding long-term oncological outcomes such as recurrence or survival. In addition, the integration of multimodal analgesia in all groups makes it difficult to isolate the sole effect of ESP block. Nonetheless, the consistency between clinical, biochemical, and metabolomic findings enhances the robustness of our conclusions.

In summary, the ESP block appears to be a safe, effective, and potentially immunomodulatory analgesic technique in breast cancer surgery. By reducing pro-tumorigenic signals and altering metabolomic profiles associated with inflammation and angiogenesis, ESP block may offer an added layer of biological protection during the perioperative period. These findings encourage the continued exploration of anesthetic strategies not only for pain control but also as modulators of the tumor–immune interface. Future prospective, randomized, and long-term studies are needed to assess the oncological implications of these perioperative interventions and to develop tailored anesthesia plans that optimize both surgical and immunological outcomes in cancer care.

## Figures and Tables

**Table 1:** Demographic data of participants.

	Morphine (n = 29)	ESP (n = 29)	Control (n = 29)	*p Value*
Age (year), mean ± SD	51.20 ± 9.60	50.83 ± 10.70	54.13 ± 9.95	*0.39 ^a^*
Body weight (kg), mean ± SD	74.26 ± 11.6	72.86 ± 10.64	70.46 ± 8.93	*0.90 ^a^*
Height (cm), mean ± SD	160.5 ± 8.32	161.7± 9.33	159.8 ± 11.27	*0.29 ^a^*
BMI (kg/m^2^), mean ± SD	27.91 ± 3.10	27.79 ± 3.63	28.712 ± 2.96	*0.50 ^a^*
ASA classification, n (%)				
I	8 (27.5)	9 (31.1)	7 (24.2)	*0.42 ^b^*
II	21 (72.5)	20 (68.9)	22 (73.8)	
Comorbidities	16 (55.1)	17 (58.6)	15 (51.7)	*0.9 ^a^*
Surgery type, n (%)				
Segmental mastectomy	21 (72.4)	18 (62.0)	17 (58.6)	*0.50 ^a^*
Modified radical mastectomy	8 (27.5)	11 (37.9)	12 (41.3)	*0.50 ^a^*

n: number, SD: standard deviation, BMI: body mass index, ASA: American Society of Anesthesiologists, ^a^: one-way ANOVA, ^b^: Pearson chi-square.

**Table 2:** Analgesic outcomes of ESP, morphine, and control groups.

Numeric Rating Scale (0–10)	Morphine (n = 29)	ESP (n = 29)	Control (n = 29)	*p*
Preoperative (mean ± SD)	0.5 ± 0.72	0.4 ± 0.56	0.7 ± 0.64	*0.16 ^a^*
Postop hour 1 (mean ± SD)	3.37 ± 0.66	1.6 ± 0.56	3.65 ± 0.72	*<0.01 ^a^*
Postop hour 2 (mean ± SD)	3.06 ± 0.69	1.53 ± 0.57	3.89 ± 0.85	*<0.01 ^a^*
Postop hour 12 (mean ± SD)	2.3 ± 0.65	2.63 ± 0.66	3.103 ± 0.77	*<0.01 ^a^*
Postop hour 24 (mean ± SD)	2.66 ± 0.71	2.8 ± 0.71	2.53 ± 0.93	*0.43 ^a^*
Opioid consumption (mg/24 h)				
Morphine (mean ± SD)	11.1 ± 4.1	N/A	N/A	*N/A*
Tramadol (mean ± SD)	N/A	57 ± 38	166 ± 69	*<0.01 ^b^*

n: number, SD: standard deviation, N/A: non-applicable, ^a^: one-way ANOVA, ^b^: Student’s *t*-test.

**Table 3:** VEGF, cortisol, and CRP levels of group morphine, ESP, and control.

	Morphine (n = 29)	ESP (n = 29)	Control (n = 29)	*p*
**VEGF (pg/mL), mean ± SD**				
T0	1219 ± 634	1092 ± 516	1312 ± 722	*0.42 ^a^*
T24	1373 ± 627	1191 ± 518	1656 ± 845	*0.025 ^b^*
**Cortisol (mcg/dL), mean ± SD**				
T0	13.6 ± 6.2	14.6 ± 6.4	14.1 ± 6.6	*0.74 ^a^*
T1	21.2 ± 10.1	19.3 ± 12.2	23.7 ± 8.1	*0.35 ^a^*
T24	19.3 ± 9.1	14.2 ± 6.1	20.0 ± 8.3	*0.041 ^b^*
**CRP (mg/L), mean ± SD**				
T0	3.8 ± 4.0	2.2 ± 1.9	2.9 ± 3.0	*0.36 ^a^*
T1	4.5 ± 5.6	1.7 ± 1.5	2.3 ± 2.0	*0.028 ^c^*
T24	29.2 ± 17.8	19.3 ± 10.7	18.3 ± 12.1	*0.022 ^a^*

n: number, SD: standard deviation, VEGF: vascular endothelial growth factor, CRP: C-reactive protein, T0: preoperative, T1: postoperative hour 1, T24: postoperative hour 24, ^a^: one-way ANOVA, ^b^: Student’s *t*-test between group ESP and control. ^c^: Student’s *t*-test between group ESP and morphine.

**Table 4:** List of metabolites that differ significantly between groups ESP and morphine.

Metabolite	Log_2_ FC	*p*-Value	On ESP Group	Biological Class	Biological Sub-Class
Homogentisic acid	2.50	1.58 × 10^−2^	↑	Benzene and substituted derivatives	Phenylacetic acids
Benzamide	10.63	3.22 × 10^−17^	↑	Benzene and substituted derivatives	Benzoic acid derivative; elevated in inflammation
Creatine	10.34	1.32 × 10^−16^	↓	Carboxylic acids and derivatives	Energy and muscle metabolism; altered in stress and inflammation
Glutathione	5.06	4.80 × 10^−6^	↑	Carboxylic acids and derivatives	Amino acids, peptides, and analogs
L-Tyrosine	3.82	4.44 × 10^−4^	↑	Carboxylic acids and derivatives	Amino acids, peptides, and analogs
Sarcosine	7.24	3.32 × 10^−10^	↓	Carboxylic acids and derivatives	Amino acids, peptides, and analogs
Leukotriene C4	6.97	1.15 × 10^−9^	↓	Carboxylic acids and derivatives	Amino acids, peptides, and analogs
N-Formyl-L-glutamic acid	5.62	4.65 × 10^−7^	↓	Carboxylic acids and derivatives	Amino acids, peptides, and analogs
S-(PGA1)-glutathione	3.75	5.46 × 10^−4^	↑	Carboxylic acids and derivatives	Amino acids, peptides, and analogs
Isobutyryl-CoA	5.55	6.24 × 10^−7^	↑	Fatty Acyls	Fatty acyl thioesters
Acrylyl-CoA	8.06	7.17 × 10^−12^	↓	Fatty Acyls	Fatty acyl thioesters
3-Oxotetradecanoyl-CoA	3.65	7.56 × 10^−4^	↓	Fatty Acyls	Fatty acyl thioesters
Palmitoylcarnitine	5.83	1.87 × 10^−7^	↑	Fatty Acyls	Pyrimidine ribonucleotides
Octanoylcarnitine	5.31	1.68 × 10^−6^	↓	Fatty Acyls	Fatty acid esters
3-Hydroxy-OPC6-CoA	5.06	4.77 × 10^−6^	↑	Fatty Acyls	Fatty acid esters
3-Methylthiopropionic acid	4.40	6.02 × 10^−5^	↑	Fatty Acyls	Fatty acids and conjugates
Prostaglandin D2	3.83	4.19 × 10^−4^	↓	Fatty Acyls	Prostaglandins and related compounds
LysoPC(O-18:0/0:0)	4.79	1.37 × 10^−5^	↑	Glycerophospholipids	Glycerophosphocholines
LysoPC(18:1/0:0)	7.36	1.95 × 10^−10^	↑	Glycerophospholipids	Glycerophosphocholines
6-Hydroxyhexanoic acid	6.71	3.86 × 10^−9^	↑	Hydroxy acids	Medium-chain hydroxy acids and derivatives
Xanthine	2.21	2.88 × 10^−2^	↑	Imidazopyrimidines	Purines and purine derivative
2-Oxoarginine	5.32	1.67 × 10^−6^	↓	Keto acids and derivatives	Short-chain keto acids and derivatives
2-Acetolactate	3.12	3.51 × 10^−3^	↓	Keto acids and derivatives	Short-chain keto acids and derivatives
Sphingosine	2.58	1.32 × 10^−2^	↑	Organonitrogen compounds	Amines
Putrescine	4.16	1.41 × 10^−4^	↑	Organonitrogen compounds	Amines
2-Phospho-D-glyceric acid	7.03	8.83 × 10^−10^	↑	Organooxygen compounds	Carbohydrates and carbohydrate conjugates
2,5-Diamino-6-(5′-triphosphoryl-3′,4′-trihydroxy-2′-oxopentyl)-amino-4-oxopyrimidine	2.43	1.82 × 10^−2^	↑	Organooxygen compounds	Carbohydrates and carbohydrate conjugates
cis-Melilotoside	4.22	1.13 × 10^−4^	↑	Organooxygen compounds	Carbohydrates and carbohydrate conjugates
3-Dehydrosphinganine	4.29	8.93 × 10^−5^	↑	Organooxygen compounds	Quinone and hydroquinone lipids
Quinone	3.60	8.79 × 10^−4^	↑	Organooxygen compounds	Quinone and hydroquinone lipids
Normetanephrine	3.35	1.88 × 10^−3^	↑	Phenols	Catecholamine metabolite; reflects sympathetic activation
Chlordecone alcohol	3.02	4.57 × 10^−3^	↑	Prenol lipids	Monoterpenoids
Perillic acid	5.73	2.88 × 10^−7^	↑	Prenol lipids	Monoterpenoids
Vitamin K1	5.48	8.51 × 10^−7^	↓	Prenol lipids	Quinone and hydroquinone lipids
Deoxyadenosine	2.98	5.07 × 10^−3^	↓	Purine nucleosides	Purine 2′-deoxyribonucleosides
Inosine triphosphate	3.44	1.44 × 10^−3^	↓	Purine nucleotides	Purine ribonucleotides
Phosphoribosyl-ATP	3.65	7.43 × 10^−4^	↑	Purine nucleotides	Purine ribonucleotides
4-Pyridoxic acid	9.62	4.20 × 10^−15^	↑	Pyridines and derivatives	Pyridinecarboxylic acids and derivatives, Vitamin B6 catabolite; oxidative stress marker
Orotidylic acid	6.55	7.78 × 10^−9^	↓	Pyrimidine nucleotides	
Taurocholic acid	4.08	1.82 × 10^−4^	↑	Steroids and steroid derivatives	Bile acid conjugate; immunological relevance
Chenodeoxycholic acid glycine conjugate	11.31	1.16 × 10^−18^	↓	Steroids and steroid derivatives	Bile acids, alcohols, and derivatives, Immune-modulatory bile acid; anti-inflammatory role
Glycocholic acid	5.25	2.20 × 10^−6^	↑	Steroids and steroid derivatives	Bile acids, alcohols, and derivatives
7 alpha,26-Dihydroxy-4-cholesten-3-one	2.80	8.03 × 10^−3^	↑	Steroids and steroid derivatives	Bile acids, alcohols, and derivatives
Taurochenodesoxycholic acid	9.85	1.38 × 10^−15^	↑	Steroids and steroid derivatives	Secondary bile acid; immune and hormonal modulation
20alpha-Dihydroprogesterone	3.93	3.10 × 10^−4^	↑	Steroids and steroid derivatives	Progesterone metabolite; anti-inflammatory effects
21-Deoxycortisol	2.75	8.96 × 10^−3^	↓	Steroids and steroid derivatives	Pregnane steroids
3a-Hydroxy-5b-pregnane-20-one	3.45	1.38 × 10^−3^	↑	Steroids and steroid derivatives	Pregnane steroids
Testosterone glucuronide	3.31	2.10 × 10^−3^	↓	Steroids and steroid derivatives	Steroidal glycosides
2-Methoxy-estradiol-17b 3-glucuronide	4.81	1.25 × 10^−5^	↓	Steroids and steroid derivatives	Steroidal glycosides
Estradiol	10.97	6.13 × 10^−18^	↑	Steroids and steroid derivatives	Estrane steroids, Steroid hormone; related to hormone-sensitive breast cancer
7a,12a-Dihydroxy-5a-cholestan-3-one	2.57	1.37 × 10^−2^	↓	Steroids and steroid derivatives	Cholestane steroids
Coproporphyrin I	3.70	6.45 × 10^−4^	↓	Tetrapyrroles and derivatives	Porphyrins
Coproporphyrin III	2.75	9.07 × 10^−3^	↓	Tetrapyrroles and derivatives	Porphyrins
Heme	9.12	4.64 × 10^−14^	↓	Tetrapyrroles and derivatives	Porphyrin-related; linked to ferroptosis and oxidative stress
Ecgonine methyl ester	4.78	1.43 × 10^−5^	↑	Tropane alkaloids	Fatty acid esters
Se-Adenosylselenohomocysteine	2.38	2.04 × 10^−2^	↑	5′-deoxyribonucleosides	

ESP: erector spinae plane block, FC: fold change ↑: increase, ↓: decrease

## Data Availability

The data supporting the findings of this study are not publicly available due to privacy and confidentiality considerations but may be obtained from the corresponding author upon reasonable request.

## Supplementary Materials

The following supporting information can be downloaded at: https://www.mdpi.com/article/10.3390/medicina61071294/s1.

## Abbreviations

The following abbreviations are used in this manuscript:

BMI	Body mass index
ASA	American Society of Anesthesiologists
ESP	Erector spinae plane block
VEGF	Vascular endothelial growth factor
CRP	C-reactive protein
PCA	Patient-controlled analgesia
Q-TOF-LC/MS	Quadrupole Time-of-Flight Mass Spectrometer
SD	Standard deviation

## References

[1] Sung H., Ferlay J., Siegel R.L., Laversanne M., Soerjomataram I., Jemal A., Bray F. (2021). Global cancer statistics 2020: GLOBOCAN estimates of incidence and mortality worldwide for 36 cancers in 185 countries. CA Cancer J. Clin..

[2] Czajka M.L., Pfeifer C. (2022). Breast Cancer Surgery. StatPearls.

[3] Coffey J.C., Wang J.H., Smith M.J., Bouchier-Hayes D., Cotter T.G., Redmond H.P. (2003). Excisional surgery for cancer cure: Therapy at a cost. Lancet Oncol..

[4] Futami R., Miyashita M., Nomura T., Makino H., Matsutani T., Sasajima K., Tajiri T. (2007). Increased serum vascular endothelial growth factor following major surgical injury. J. Nippon. Med. Sch..

[5] Neeman E., Zmora O., Ben-Eliyahu S. (2012). A New Approach to Reducing Postsurgical Cancer Recurrence: Perioperative Targeting of Catecholamines and Prostaglandins. Clin. Cancer Res..

[6] Hiller J.G., Perry N.J., Poulogiannis G., Riedel B., Sloan E.K. (2018). Perioperative events influence cancer recurrence risk after surgery. Nat. Rev. Clin. Oncol..

[7] Neeman E., Ben-Eliyahu S. (2013). Surgery and stress promote cancer metastasis: New outlooks on perioperative mediating mechanisms and immune involvement. Brain Behav. Immun..

[8] Hayes D.F., Cristofanilli M., Budd G.T., Ellis M.J., Stopeck A., Miller M.C., Matera J., Allard W.J., Doyle G.V., Terstappen L.W. (2006). Circulating Tumor Cells at Each Follow-Up Time Point During Therapy of Metastatic Breast Cancer Patients Predict Progression-Free and Overall Survival. Clin. Cancer Res..

[9] Gottschalk A., Sharma S., Ford J., Durieux M.E., Tiouririne M. (2010). The Role of the Perioperative Period in Recurrence After Cancer Surgery. Anesth. Analg..

[10] Ben-Eliyahu S. (2003). The promotion of tumor metastasis by surgery and stress: Immunological basis and implications for psychoneuroimmunology. Brain Behav. Immun..

[11] Wall T., Sherwin A., Ma D., Buggy D.J. (2019). Influence of perioperative anaesthetic and analgesic interventions on oncological outcomes: A narrative review. Br. J. Anaesth..

[12] Desborough J. (2000). The stress response to trauma and surgery. Br. J. Anaesth..

[13] Hanahan D., Weinberg R.A. (2011). Hallmarks of cancer: The next generation. Cell.

[14] Baxevanis C.N., Papilas K., Dedoussis G.V., Pavlis T., Papamichail M. (1994). Abnormal cytokine serum levels correlate with impaired cellular immune responses after surgery. Clin. Immunol. Immunopathol..

[15] Zhao T., Xia W., Zheng M., Lu C., Han X., Sun Y. (2008). Surgical excision promotes tumor growth and metastasis by promoting expression of MMP-9 and VEGF in a breast cancer model. Exp. Oncol..

[16] Calvo-Soto P., Martíanez-Contreras A., Trujillo-Hernández B., Peraza-Garay F., Vásquez C. (2012). Spinal—General anaesthesia decreases neuroendocrine stress response in laparoscopic cholecystectomy. J. Int. Med. Res..

[17] Matsubara D., Arita T., Nakanishi M., Kuriu Y., Murayama Y., Kudou M., Konishi H., Komatsu S., Shiozaki A., Otsuji E. (2020). The impact of postoperative inflammation on recurrence in patients with colorectal cancer. Int. J. Clin. Oncol..

[18] Muthubharathi B.C., Gowripriya T., Balamurugan K. (2021). Metabolomics: Small molecules that matter more. Mol. Omics.

[19] Mock-Ohnesorge J., Mock A., Hackert T., Fröhling S., Schenz J., Poschet G., Jäger D., Büchler M.W., Uhle F., Weigand M.A. (2021). Perioperative changes in the plasma metabolome of patients receiving general anesthesia for pancreatic cancer surgery. Oncotarget.

[20] Maniwa Y., Okada M., Ishii N., Kiyooka K. (1998). Vascular endothelial growth factor increased by pulmonary surgery accelerates the growth of micrometastases in metastatic lung cancer. Chest.

[21] Pang Z., Chong J., Zhou G., de Lima Morais D.A., Chang L., Barrette M., Gauthier C., Jacques P.-É., Li S., Xia J. (2021). MetaboAnalyst 5.0: Narrowing the gap between raw spectra and functional insights. Nucleic Acids Res..

[22] Veronesi U., Cascinelli N., Mariani L., Greco M., Saccozzi R., Luini A., Aguilar M., Marubini E. (2002). Twenty-year follow-up of a randomized study comparing breast-conserving surgery with radical mastectomy for early breast cancer. N. Engl. J. Med..

[23] Li S., Yan W., Yang X., Chen L., Fan L., Liu H., Liu K., Zhang Y., Jiang J. (2019). Less micrometastatic risk related to circulating tumor cells after endoscopic breast cancer surgery compared to open surgery. BMC Cancer.

[24] Tagliabue E., Agresti R., Carcangiu M.L., Ghirelli C., Morelli D., Campiglio M., Martel M., Giovanazzi R., Greco M., Balsari A. (2003). Role of HER2 in wound-induced breast carcinoma proliferation. Lancet.

[25] Demicheli R., Desmedt C., Retsky M., Sotiriou C., Piccart M., Biganzoli E. (2020). Late effects of adjuvant chemotherapy adumbrate dormancy complexity in breast cancer. Breast.

[26] Choi H., Hwang W. (2024). Anesthetic Approaches and Their Impact on Cancer Recurrence and Metastasis: A Comprehensive Review. Cancers.

[27] Exadaktylos A.K., Buggy D.J., Moriarty D.C., Mascha E., Sessler D.I. (2006). Can anesthetic technique for primary breast cancer surgery affect recurrence or metastasis?. Anesthesiology.

[28] Lucchinetti E., Awad A.E., Rahman M., Feng J., Lou P.H., Zhang L., Ionescu L., Lemieux H., Thébaud B., Zaugg M. (2012). Antiproliferative effects of local anesthetics on mesenchymal stem cells: Potential implications for tumor spreading and wound healing. Anesthesiology.

[29] Hönemann C.W., Heyse T.J., Möllhoff T., Hahnenkamp K., Berning S., Hinder F., Linck B., Schmitz W., van Aken H. (2001). The inhibitory effect of bupivacaine on prostaglandin E(2) (EP(1)) receptor functioning: Mechanism of action. Anesth. Analg..

[30] Gürkan Y., Aksu C., Kuş A., Yörükoğlu U.H., Kılıç C.T. (2018). Ultrasound guided erector spinae plane block reduces postoperative opioid consumption following breast surgery: A randomized controlled study. J. Clin. Anesth..

[31] Jafra A., Sharma S., Arora S., Singh G. (2020). Efficacy of erector spinae plane block for postoperative analgesia in total mastectomy and axillary clearance: A randomized controlled trial. Saudi J. Anaesth..

[32] Rocha-Romero A., Fajardo-Perez M. (2021). Function of the sympathetic supply in the erector spinae plane block. Can. J. Anaesth..

[33] Hu Y., Li M., Li J., Lyu Q., Jiang R., Du Y. (2021). Effects of ultrasound-guided erector spinae plane block on the immune function and postoperative recovery of patients undergoing radical mastectomy. Gland. Surg..

[34] Ali E.M., Sheta M., El Mohsen M.A. (2011). Elevated serum and tissue VEGF associated with poor outcome in breast cancer patients. Alex. J. Med..

[35] Neufeld G., Cohen T., Gengrinovitch S., Poltorak Z. (1999). Vascular endothelial growth factor (VEGF) and its receptors. FASEB J..

[36] Ohm J.E., Carbone D.P. (2001). VEGF as a mediator of tumor-associated immunodeficiency. Immunol. Res..

[37] Sproston N.R., Ashworth J.J. (2018). Role of C-Reactive Protein at Sites of Inflammation and Infection. Front. Immunol..

[38] Ito K., Yoshii H., Sato A., Kuroda K., Asakuma J., Horiguchi A., Sumitomo M., Asano T. (2011). Impact of Postoperative C-Reactive Protein Level on Recurrence and Prognosis in Patients with N0M0 Clear Cell Renal Cell Carcinoma. J. Urol..

[39] Butterworth J., James R.L., Grimes J. (1997). Structure-affinity relationships and stereospecificity of several homologous series of local anesthetics for the beta2-adrenergic receptor. Anesth. Analg..

[40] Hellstrand K., Czerkinsky C., Ricksten A., Jansson B., Asea A., Kylefjord H., Hermodsson S. (1993). Role of serotonin in the regulation of interferon-gamma production by human natural killer cells. J. Interf. Res..

[41] Saeed I., La Caze A., Hollmann M.W., Shaw P.N., Parat M.-O. (2021). New Insights on Tramadol and Immunomodulation. Curr. Oncol. Rep..

[42] Finnerty C.C., Mabvuure N.T., Ali A., Kozar R.A., Herndon D.N. (2013). The surgically induced stress response. J. Parenter Enter. Nutr..

[43] Buyukkocak U., Daphan C., Caglayan O., Aydinuraz K., Kaya T., Saygun O., Agalar F. (2006). Effects of different anesthetic techniques on serum leptin, C-reactive protein, and cortisol concentrations in anorectal surgery. Croat. Med. J..

[44] Shirzad H., Shahrani M., Rafieian-Kopaei M. (2009). Comparison of morphine and tramadol effects on phagocytic activity of mice peritoneal phagocytes in vivo. Int. Immunopharmacol..

[45] Cheng S., Guo M., Liu Z., Fu Y., Wu H., Wang C., Cao M. (2019). Morphine Promotes the Angiogenesis of Postoperative Recurrent Tumors and Metastasis of Dormant Breast Cancer Cells. Pharmacology.

[46] Gupta K., Kshirsagar S., Chang L., Schwartz R., Law P.-Y., Yee D., Hebbel R.P. (2002). Morphine stimulates angiogenesis by activating proangiogenic and survival-promoting signaling and promotes breast tumor growth. Cancer Res..

[47] Bortsov A.V., Millikan R.C., Belfer I., Boortz-Marx R.L., Arora H., McLean S.A. (2012). μ-Opioid receptor gene A118G polymorphism predicts survival in patients with breast cancer. J. Am. Soc. Anesthesiol..

[48] Sacerdote P., Bianchi M., Manfredi B., E Panerai A. (1997). Effects of tramadol on immune responses and nociceptive thresholds in mice. Pain.

[49] Tsai Y.C., Won S.J. (2001). Effects of tramadol on T lymphocyte proliferation and natural killer cell activity in rats with sciatic constriction injury. Pain.

[50] Kim M.H., Lee J.-R., Kim K.-J., Jun J.H., Hwang H.J., Lee W., Nam S.H., Oh J.E., Yoo Y.C. (2021). Identification for antitumor effects of tramadol in a xenograft mouse model using orthotopic breast cancer cells. Sci. Rep..

[51] Sacerdote P., Bianchi M., Gaspani L., Manfredi B., Maucione A., Terno G., Ammatuna M., Panerai A.E. (2000). The Effects of Tramadol and Morphine on Immune Responses and Pain After Surgery in Cancer Patients. Anesth. Analg..

[52] Fu J., Yu M., Xu W., Yu S. (2022). Research progress of bile acids in cancer. Front. Oncol..

[53] Duboc H., Rajca S., Rainteau D., Benarous D., Maubert M.-A., Quervain E., Thomas G., Barbu V., Humbert L., Despras G. (2013). Connecting dysbiosis, bile-acid dysmetabolism and gut inflammation in inflammatory bowel diseases. Gut.

[54] Fu X.-D., Garibaldi S., Gopal S., Polak K., Palla G., Spina S., Mannella P., Genazzani A.D., Simoncini T. (2011). Dydrogesterone exerts endothelial anti-inflammatory actions decreasing expression of leukocyte adhesion molecules. Mol. Hum. Reprod..

[55] Sanderson J.T. (2006). The Steroid Hormone Biosynthesis Pathway as a Target for Endocrine-Disrupting Chemicals. Toxicol. Sci..

[56] Capper C.P., Rae J.M., Auchus R.J. (2016). The Metabolism, Analysis, and Targeting of Steroid Hormones in Breast and Prostate Cancer. Discov. Oncol..

[57] Li M., Li J. (2024). Steroids in Cancer: Mechanisms, Therapies, and Challenges in Hormone-Driven Malignancies.

[58] Acconcia F., Marino M., Belfiore A., LeRoith D. (2016). Steroid Hormones: Synthesis, Secretion, and Transport. Principles of Endocrinology and Hormone Action.

[59] Dutra F.F., Bozza M.T. (2014). Heme on innate immunity and inflammation. Front. Pharmacol..

[60] Aftab H., Donegan R.K. (2024). Regulation of heme biosynthesis via the coproporphyrin dependent pathway in bacteria. Front. Microbiol..

[61] Consoli V., Sorrenti V., Pittalà V., Greish K., D’amico A.G., Romeo G., Intagliata S., Salerno L., Vanella L. (2022). Heme oxygenase modulation drives ferroptosis in TNBC cells. Int. J. Mol. Sci..

[62] Bronte V., Zanovello P. (2005). Regulation of immune responses by L-arginine metabolism. Nat. Rev. Immunol..

[63] Page T.H., Smolinska M., Gillespie J., Urbaniak A.M., Foxwell B.M. (2009). Tyrosine kinases and inflammatory signalling. Curr. Mol. Med..

[64] Yang F., Xiao Y., Ding J.-H., Jin X., Ma D., Li D.-Q., Shi J.-X., Huang W., Wang Y.-P., Jiang Y.-Z. (2022). Ferroptosis heterogeneity in triple-negative breast cancer reveals an innovative immunotherapy combination strategy. Cell Metab..

[65] Didžiapetrienė J., Kazbarienė B., Tikuišis R., Dulskas A., Dabkevičienė D., Lukosevičienė V., Kontrimavičiūtė E., Sužiedėlis K., Ostapenko V. (2020). Oxidant/Antioxidant Status of Breast Cancer Patients in Pre- and Post-Operative Periods. Medicina.

